# Long-term and daily use of molecular hydrogen induces reprogramming of liver metabolism in rats by modulating NADP/NADPH redox pathways

**DOI:** 10.1038/s41598-022-07710-6

**Published:** 2022-03-10

**Authors:** Yao Mawulikplimi Adzavon, Fei Xie, Yang Yi, Xue Jiang, Xiaokang Zhang, Jin He, Pengxiang Zhao, Mengyu Liu, Shiwen Ma, Xuemei Ma

**Affiliations:** 1 Faculty of Environment and Life, Beijing University of Technology, Beijing, 100124 People’s Republic of China; 2 Beijing Molecular Hydrogen Research Center, Beijing, 100124 People’s Republic of China

**Keywords:** Biochemistry, Molecular biology, Physiology, Molecular medicine

## Abstract

Molecular hydrogen (H_2_) has emerged as a new therapeutic option in several diseases and is widely adopted by healthy people. However, molecular data to support therapeutic functions attributed to the biological activities of H_2_ remain elusive. Here, using transcriptomic and metabolomic approaches coupled with biochemistry and micro-CT technics, we evaluated the effect of long-term (6 months) and daily use of H_2_ on liver function. Rats exposed 2 h daily to H_2_ either by drinking HRW (H_2_ dissolved in H_2_O) or by breathing 4% H_2_ gas showed reduced lipogenesis and enhanced lipolysis in the liver, which was associated with apparent loss of visceral fat and brown adipose tissue together with a reduced level of serum lipids. Both transcripts and metabolites enriched in H_2_-treated rats revealed alteration of amino acid metabolism pathways and activation of purine nucleotides and carbohydrate biosynthesis pathways. Analysis of the interaction network of genes and metabolites and correlation tests revealed that NADP is the central regulator of H_2_ induced metabolic alterations in the liver, which was further confirmed by an increase in the level of components of metabolic pathways that require NADP as substrate. Evidence of immune response regulation activity was also observed in response to exposure to H_2_. This work is the first to provide metabolomic and transcriptomic data to uncover molecular targets for the effect of prolonged molecular hydrogen treatment on liver metabolism.

## Introduction

Molecular hydrogen (H_2_) is a tasteless and odorless gas and has demonstrated various biological and therapeutic effects on many diseases, from acute illnesses, including ischemia–reperfusion injury, to chronic diseases such as rheumatoid arthritis, neurodegenerative, and metabolic diseases^1–3^. H_2_ is non-toxic even when used at high concentrations and rapidly crosses different tissue barriers, including blood–brain barriers, and penetrates various organelles^4–6^. H_2_ is interferes with reactive oxygen species (ROS) in living systems, a characteristic that supports its well-established antioxidant functions that lead to its frequent use to treat diseases associated with oxidative stress^3,7,8^.

Molecular hydrogen is applied in therapeutics through various delivery routes, including hydrogen inhalation (HI), oral administration of hydrogen-rich water (HRW) or hydrogen tablets, and hydrogen-saturated saline injections. The concentration of H_2_ in tissues after exogenous supply depends on the type of organ^4^ and the H_2_ delivery route^9^. Liver is the organ that accumulates more exogenous H_2_^4,9^, which appears to significantly affect its homeostasis^10^. H_2_ protected the liver against various acute and chronic injuries in animal models by suppressing excessive oxidative stress, inflammation, and cell death^11,12^. In addition to liver, preventive and therapeutic effects were obtained for H_2_ in various animal experiments of disease models^2,3^ and clinical trials in humans^13–15^. The preventive aspect of H_2_ in disease models, its antioxidant effects^7,8^, its function in aging^13,16^, among many others^17–19^, are paving the way for the use of H_2_ in healthy people to improve body performance or as a preventive therapeutic strategy.

In a recent clinical trial that was evaluated in a cohort of young and healthy people, the effects of inhaling 4% H_2_ 20 min per day for 7 days on exercise performance revealed ergogenic properties such as improved running performance and torso strength^20^. Although Sim M. and colleagues had reported the beneficial effect of H_2_ inhalation on the increase of the antioxidant and anti-inflammatory response in healthy adults^21^, other groups found that H_2_ only reduced delayed-onset muscle soreness after running downhill^22^ or improved muscle function during exercise without any effect on blood oxidative markers^23^. These data suggest that still little is known about H_2_ performance under healthy conditions and therefore requires further research. As part of an effort to provide more data to help better understand the therapeutic functions attributed to the biological activities of H_2_ in health conditions, our laboratory has initiated a study that evaluated the effect of long-term (6 months) hydrogen intervention on the physiological function of healthy rats^24^. The study found that H_2_ induces a time-dependent alteration of different biochemical parameters, among which liver injury markers such as alanine aminotransferase (ALT), aspartate aminotransferase (AST), and total bile acid (TBA) were significantly reduced in the serum of H_2_-treated rats^24^, suggesting that liver physiology and functions could be positively affected by prolonged exposure to H_2_. In the present study, we used transcriptomic and metabolomic approaches coupled with biochemistry and micro-CT techniques to access the global effects of long-term H_2_ treatment on the liver and its relationship with body conditions.

## Materials and methods

### Animals and experimental design

Three-week-old male Sprague-Dawley rats weighing 40–50 g were purchased from Vital River Laboratory Animal Technology Co., Ltd (Beijing, China). The animals were kept under standard conditions at 22 °C to 25 °C with a 12 h light–dark cycle and fed a normal diet. The animals were allowed to adapt to laboratory conditions one week before the experiment. Rats were randomly divided into three groups and treated with or without H_2_ for six months. Rats in the control group (n = 6) were kept under normal conditions; rats in the HRW group (n = 6) were given access to HRW for 1 h, twice a day; In the HI group (n = 6), rats were exposed to 4% hydrogen gas for 1 h and twice a day. All procedures were approved by the Institutional Animal Experiment Committee of the Chinese People's Liberation Army (PLA) General Hospital and were carried out in accordance with the Regulations for the Administration of Affairs Concerning Experimental Animals (China) and the ARRIVE guidelines.

### Hydrogen-rich water preparation

HRW (H_2_ concentration > 800 µM) was kindly provided by Shenzhen Kelieng Biomedical Co. Ltd. (Shenzhen, China) and stored under atmospheric pressure at 23 ± 2 °C in a stainless-steel bucket (KLE-8). The hydrogen concentration was monitored using a hydrogen electrode (Unisense A/S, Aarhus, Denmark), ensuring that the hydrogen concentration of HRW for rats was maintained above 800 µM.

### Inhalation of hydrogen gas

In the HI experiments, the rats were placed in breathing boxes (72 × 53 × 45 cm, length × width × height), where a gas mixture of 4% H_2_ and 96% air containing 21% O_2_ were delivered by an Oxy-Hydrogen Machine (SG-3000; Gang’an Health Management [Beijing] Co., Ltd., Beijing, China). The mixture was administered twice a day for 1 h each and, its composition was monitored using the Thermal trace GC ultragas chromatography (Thermo Fisher, MA, USA).

### Micro-computed tomography (micro-CT) analysis

After anesthesia, rats were scanned by Quantum GX microCT (PerkinElmer) with voltage at 74 kV, view imaging at 72 × 40 mm, and pixel size at 72 μm. Subcutaneous and visceral fat mass was analyzed by the software (Analyze 12.0, PerkinElmer).

### Tissue and blood sampling

Blood was sampled by eyeball blood collection early in the morning from overnight fasting rats. Serum was prepared by 20 min centrifugation at 1000 × g. Rats were then sacrificed by cervical dislocation, and the livers were harvested, snap-frozen in liquid nitrogen, and stored at − 80 °C until subsequent analysis. Intraabdominal perirenal (pWAT), epididymal (eWAT), subcutaneous inguinal (sWAT) white adipose tissue, and brown adipose tissue (BAT) were dissected and weighted.

### Biochemical analysis

Serum total cholesterol, HDL-cholesterol (HDL-C), and LDL-cholesterol (LDL-C) were determined using commercially available kits (Nanjing Jiancheng Biochemistry, China) according to the manufacturer’s instructions. The hormone-sensitive lipase (HSL) activity and the level of epinephrine in serum were evaluated using ELISA kits from MEIMIAN Industrial Co., Ltd. (Jiangsu, China).

### Transcriptomic analysis

Total RNA was extracted from liver tissues with a standard Trizol RNA extraction procedure. RNA quality was assessed using the RNA Nano 6000 Assay Kit of the Bioanalyzer 2100 system (Agilent, USA). Sequencing libraries were generated using the NEBNextR Ultra RNA Library Prep Kit for Illumina^®^ (#E7530L, NEB, USA). The size of the library insert was tested using the Agilent Bioanalyzer 2100 system (Agilent, USA). The Bio-RAD CFX 96 fluorescent quantitative PCR instrument and Bio-RAD KIT iQ SYBR GRN were used to perform Q-PCR for accurate quantification of the effective concentration of the library (effective concentration of the library > 10 nM). After cluster generation, the libraries were sequenced by the Illumina NovaSeq 6000 S4 platform with paired-end reads by Annoroad Gene Technology (Beijing, China). Differential gene expression (DEGs) analysis was performed using the DESeq2R package version 1.16.3. and the DEGs were selected with |LogFC|> 1 and FDR < 0.05.

### Untargeted metabolomics analysis

The liver sample (50 mg) was homogenized in 500 µL prechilled methanol, vortexed, and sonicated for 20 min on ice, incubated at − 20 °C for 1 h, and centrifuged at 14,000 g for 20 min at 4 °C. The extracted metabolites were concentrated by complete drying using a speedvac (Labconco, USA), redissolved in 100 µL acetonitrile–water solution (1:1, v/v), and centrifuged at 14,000 g for 20 min at 4 °C. The supernatant was sent to the Metabolomics Facility at Tsinghua University Branch of the China National Center for Protein Sciences (Beijing, China) and used for HPLC–MS or MS analysis. Untargeted metabolomics was performed using an ultra-performance liquid chromatography Q-Exactive Orbitrap mass spectrometer (UPLC Q-Exactive Orbitrap MS). The metabolomics data analysis and interpretation were performed with the MetaboAnalyst 5.0 web-based interface^25^. Significant metabolites were selected with |FC|≥ 1.5 and *p* value < 0.05.

### Annotation and network analysis

The WEB-based Gene Set Analysis Toolkit^26^ and annotation and network analysis modules in MetaboAnalyst 5.0^25^ were used to get biological insights of genes and metabolites differentially expressed, respectively. GraphPad Prism version 9.0.0 (121) was used to plot data and perform statistical analysis. One-way ANOVA with Tukey’s post hoc test was used for error correction. Data were presented as the mean ± SEM, and a *p* value < 0.05 was considered significant.

## Results

### H_2_ influences liver metabolism of lipids, carbohydrates, amino acids, and nucleic acids

**Figure 1:**
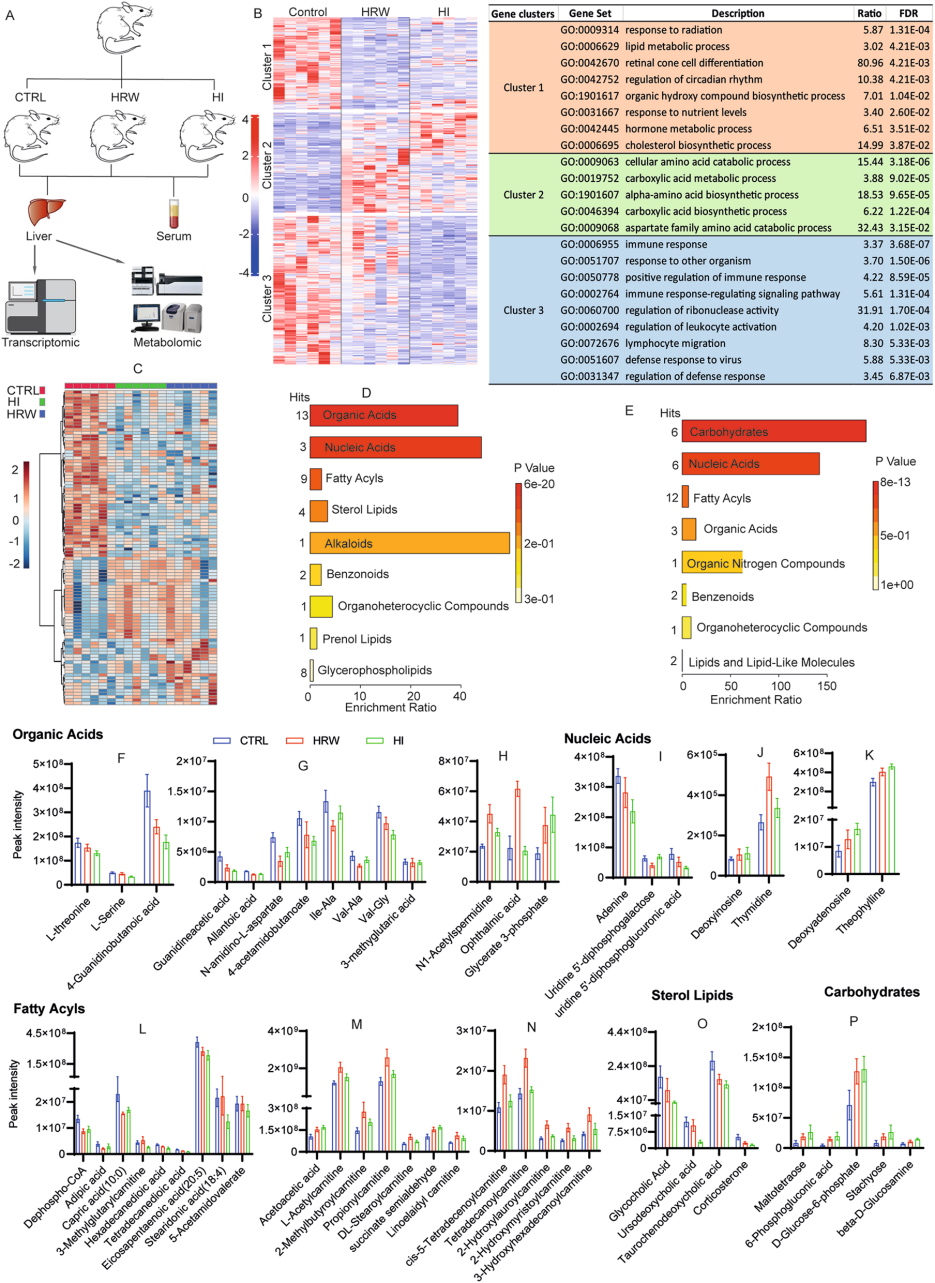
Long-term use of H_2_ induces an alteration of metabolism in the liver of healthy rats. (**A**) Diagram of experimental design and sample processing methods. (**B**) Heatmap of the DEGs and biological process ontology term for the genes in each cluster. (**C**) Heatmap of metabolites significantly altered and an overview of the enriched metabolites sets for down (**D**) and up-regulated (**E**) metabolites in H_2_ (HRW and HI) groups compared to control. (**F**–**P**) The expression level of metabolite differentially expressed compared to control (|FC|> 1.5, *p* value < 0.05) and significantly enriched in metabolites set with a *p* value < 0.05; data are plotted as Mean ± SEM, and their specific fold change (FC) and *p* value are detailed in supplementary file 2.

We recently found that H_2_-treated rats (HRW and HI) had reduced serum levels of alanine aminotransferase (ALT), aspartate aminotransferase (AST), and total bile acid (TBA)^24^, suggesting that prolonged exposure to H_2_ could influence liver physiology and functions. To study the global effects of long-term H_2_ treatment on the liver, we performed an RNA sequencing (RNA seq) analysis of liver tissues collected from untreated rats (Control group), rats given hydrogen-rich water (HRW) or inhaled 4% H_2_ (HI) 2 h daily for 6 months (Fig. 1A, B). We identified from the liver transcriptome, 828 differentially expressed genes (DEGs): 321 in HRW versus CTRL, 435 in HI versus C, and 72 in HI versus HRW, of which there were 625 unique DEGs (Supplementary file 1). Profiling the expression of these 625 DEGs in all experimental groups showed three distinct expression clusters (Fig. 1B). The genes in cluster 1, down-regulated in the HRW and HI groups, are mainly involved in biological functions related to lipid metabolism and hormone synthesis; the genes in cluster 2, up-regulated by HRW and HI, play functions in cellular amino acid catabolic and carboxylic acid biosynthesis process; The cluster 3 regrouped genes enriched in immune system-related functions, and their expression appeared to be more specific to HI (Fig. 1B, Supplementary file 1). These data suggest that H_2_ could also induce changes in liver immune functions in addition to its metabolic regulatory activities.

To further our understanding of the impact of H_2_ on liver metabolism, we considered a comparative metabolomics approach that generates a profile of over 500 metabolites from various chemical classes. We observed a significant alteration in more than 100 metabolites, including organic acids, carbohydrates, nucleic acids, fatty acyls, and sterol lipids in the liver of H_2_-treated rats compared to untreated rats (Fig. 1C–E, Supplementary file 2). A closer inspection of the organic acid groups affected by H_2_ treatment revealed a reduced level of amino acids such as L-serine, L-threonine, and Guanidineacetic acid, components of the glycine, serine and threonine metabolic pathways, and 4-Guanidinobutanoate and 4-Acetamidobutanoate, members of the arginine and proline metabolic pathways (Fig. 1F, G; Supplementary file 2).

The level of nucleoside diphosphate sugars, uridine 5'-diphosphogalactose and uridine 5'-diphosphoglucuronic acid, the two most common nucleotide sugar donors in animal cells^27^ for the biosynthesis of polysaccharides, glycoproteins, and glycolipids^28,29^, was reduced, consistent with the increased level of carbohydrates observed in H_2_ treated rats (Fig. 1I, P; Supplementary file 2). Rats treated with H_2_ also showed an altered liver level of metabolites enriched in purine metabolism pathways. These rats had reduced levels of adenine (Fig. 1I), an increased level of deoxyadenosine and deoxyinosine (Fig. 1J, K), which, together with the unchanged liver content in uric acid and its reduced serum level (Supplementary Figure1A, B), suggests that long-term exposure to H_2_ may activate purine biosynthetic pathways.

Although long-term H_2_ treatment induced a reduced level of fatty acyls and sterol lipids enriched in the biosynthesis pathways of lipids, bile acids, and steroid hormones (Fig. 1L, O; Supplementary file 2), it increases the level of acylcarnitines (Fig. 1M, N), fundamental players in the transport of organic acids and fatty acids from the cytoplasm to mitochondria^30^ for oxidation to produce energy (Supplementary file 2). Furthermore, we note that H_2_ treatment induced an increased level of acetoacetate (Fig. 1M), a ketone body produced from fatty acid oxidation^30,31^ and previously reported to increase lipolysis and decrease lipid synthesis^32^, supporting the role of H_2_ in promoting liver fatty acid oxidation.

Taken together, our data support the function of long-term use of H_2_ in inducing lipolysis, amino acid catabolism to eventually support the pathways of biosynthesis of carbohydrates and purine nucleotides.

### H_2_-induced alteration of liver metabolism is accompanied by lipolysis

**Figure 2:**
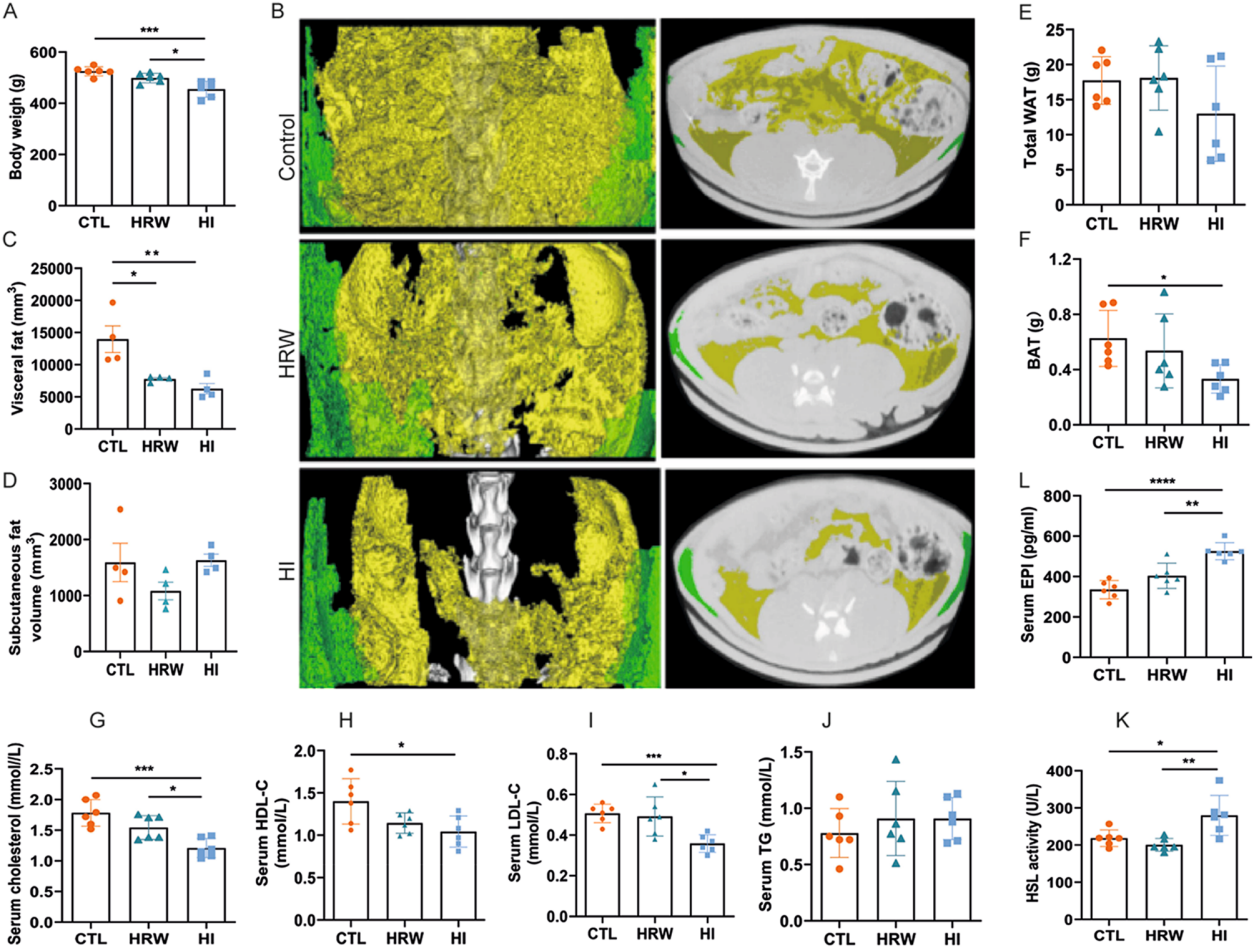
Change in body composition and serum biochemical parameters in response to long-term H_2_ intervention. (**A**) Change in body weight and body fat composition measured by micro-CT (B). (**C**–**D**) Change in the volume of subcutaneous (green in panel **B**) and visceral fat (yellow in panel **B**) quantified by the Analyze12.0 software (PerkinElmer) from the micro-CT data. Mass of total WAT and BAT are presented in (**E**) and (**F**), respectively. Graphs in (**G**–**J**) summarize our previous finding of serum lipids of rats exposed to H_2_ for 6 months^24^. The data of the level of epinephrine (**L**) in serum and the activity of HSL from rat liver lysates (**K**) are presented. Data are shown as Mean ± SEM. *p value < 0.05; **p value < 0.01; ***p value < 0.001.

Liver plays a central role in the regulation of metabolism and represents a communication bridge between organ systems^33^, suggesting that H_2_-induced alteration of liver metabolism could affect the whole-body metabolism and, therefore, body condition. To assess the effect of long-term use of H_2_ on body composition, we measured the change in body weight (Fig. 2A) and fat mass (Fig. 2B–F) and change in serum metabolites (Fig. 2G–J). Long-term H_2_ intervention reduced the body weight of rats in the HI group (455.20 ± 31.57 g), while no significant changes were observed in the HRW group (498.80 ± 18.50 g) compared to controls (525.00 ± 17.78 g) (Fig. 2A)^24^. Visceral fat volume (HRW: 7760 ± 429 mm^3^; HI: 6260 ± 1624 mm^3^; CTRL: 13,960 ± 4138 mm^3^) (Fig. 2B, C) and brown adipose tissue mass (BAT) (HRW: 0.54 ± 0.27 g; HI: 0.33 ± 0.10 g; CTRL: 0.63 ± 0.20) (Fig. 2F) were reduced in rats exposed to H_2_; white adipose tissue mass (WAT) (Fig. 2E) and subcutaneous fat (Fig. 2B, D) were unchanged. Although the total triglyceride level in serum was unchanged (Fig. 2J)^24^, we found the lipolytic stimulator epinephrine^34,35^ (HI: 525.4 ± 42.40 pg/ml, HRW: 403.8 ± 62.48 pg/ml, CTRL: 335.4 ± 45.52 pg/ml,) (Fig. 2L) and the hormone sensitive lipase HSL (HI: 279.90 ± 53.76 U/L vs. CTRL: 218.50 ± 22.28 U/L, *p* = 0.022) (Fig. 2K), high in serum and liver respectively of H_2_ treated rats compared to control groups. These data, together with our previous report^24^ on the decreased level of serum lipids, such as total cholesterol, high-density lipoprotein cholesterol (HDL-C), and low-density lipoprotein cholesterol (LDL-C), induced by long-term treatment with H_2_ (Fig. 2G–I), showed that long-term exposure to H_2_ could modulate body mass by affecting body fat composition.

### H_2_ induces metabolic alteration in liver by modulating NADP/NADPH redox pathways

**Figure 3:**
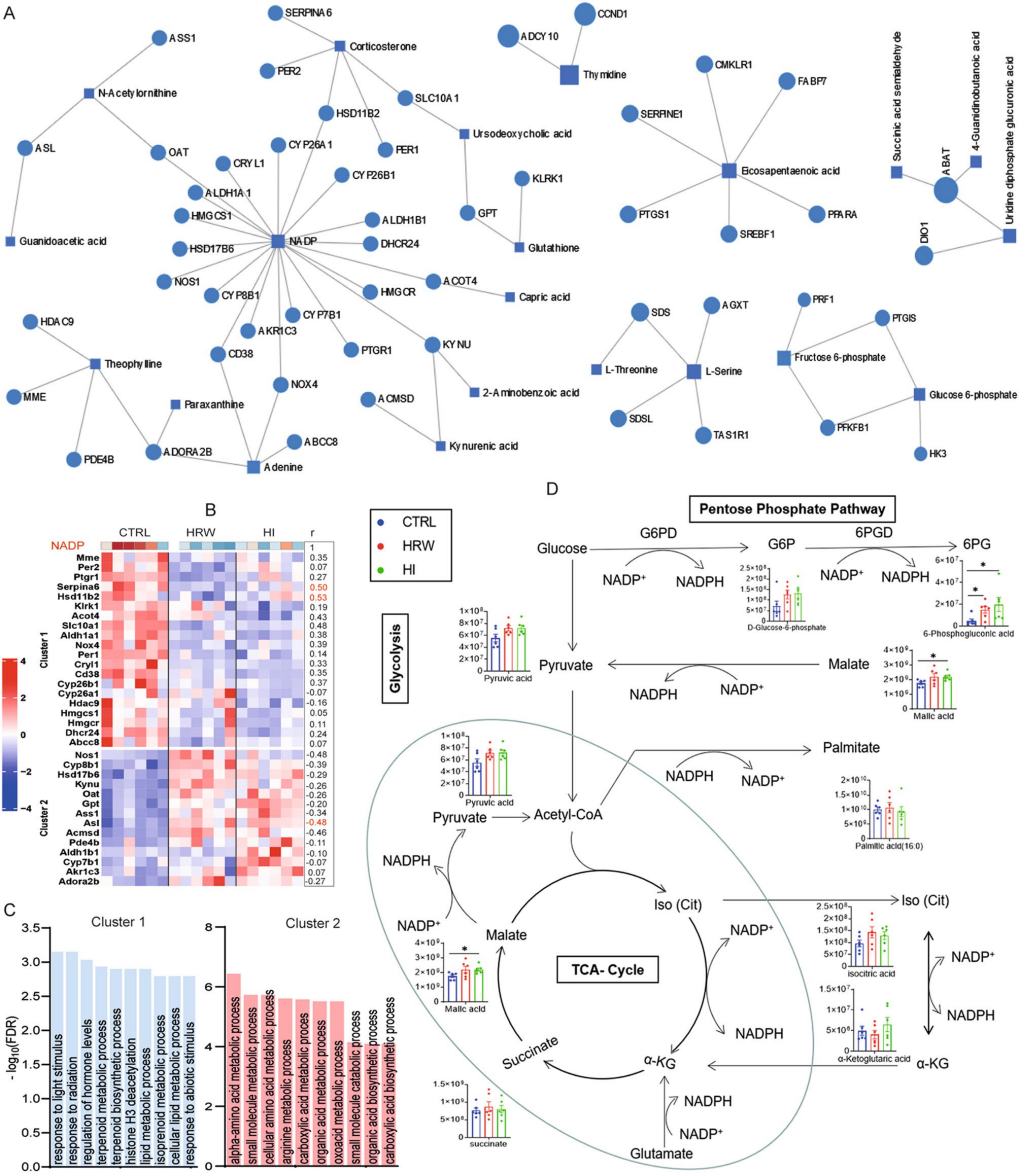
The implication of NADP/NADPH pathways in the metabolic regulatory functions of H_2_. (**A**) Genes and metabolites interaction network for differentially expressed genes and metabolites (genes and metabolites are shown in circles and squares, respectively). (**B**) Heatmap showing the expression profile of genes that encode metabolic enzymes connected to NADP. The coefficient (r) of the spearman correlation analysis is shown on the right of the heatmap. Data of statistically significant correlation results at a level of *p* value < 0.05 is shown in red. (**C**) Biological functions of genes that encode metabolic enzymes enriched in the NADP network. (**D**) Metabolic reaction of NADP/NADPH consumption and production pathways. The effect of H_2_ on the level of components of these metabolic pathways is shown. Data are presented as Mean ± SEM. *p value < 0.05; **p value < 0.01; ***p value < 0.001.

To gain further insight into the mechanism associated with the reprogramming of liver metabolism by long-term H_2_ intervention, we performed an interaction network analysis for genes and metabolites with a significant change in response to H_2_ exposure. The analysis identified six subnets of genes encoding metabolic enzymes and the corresponding metabolites (Fig. 3A, Supplementary file 3). NADP has the highest degree of node and connected metabolic enzyme-coding genes enriched in biological functions related to the metabolic process of lipids, amino acids, and carboxylic acids (Fig. 3B, C), the main alteration that we observed (Figs. 1, 2) during the H_2_ intervention. Long-term H_2_ intervention decreased the liver level of NADP (Fig. 3B, Supplementary Fig. 2) that was positively correlated with a reduced expression of genes coding for lipid and hormone metabolizing enzymes, while a negative correlation was observed for enzyme-coding genes involved in the metabolism of amino acids and carboxylic acids biosynthetic process (Fig. 3B). Knowing that the reduced form of NADP, NADPH provides high energy electrons for antioxidant defense and is necessary for nucleotide, amino acid, and lipid biosynthesis^36,37^, we hypothesized that the H_2_-induced decrease in the level of NADP would affect metabolic pathways that required NADP as the final acceptor of electrons. NADP and its reduced form NADPH are produced or consumed in the cell cytosol during reactions of cellular energy metabolism that involve glycolysis and the pentose phosphate pathway (PPP) and in the mitochondria during the tricarboxylic acid cycle (TCA)^36–40^. The NADP/NADPH pool can also be affected by metabolism reactions involving CYP450s that utilize NADPH as an electron donor^41^. Analysis of the effect of H_2_ on the level of components of these metabolic pathways showed that H_2_ treatment increased the level of metabolites such as G6P and 6PG (PPP) and malic acid (TCA cycle and cytosol) involved in reactions that reduced NADP to NADPH (Fig. 3D). However, the expression level of genes that encode cytochrome P450 oxidoreductases such as Cyp26b1, Cyp26a1, which lead to the accumulation of NADP, was found to be down-regulated after H_2_ treatment (Fig. 3B). Together, these data suggest that H_2_ induces reprogramming of liver metabolism through modulation of biological pathways that required NADP as an electron acceptor (reduction of NADP to NADPH).

## Discussion

The therapeutic potential of H_2_ has been widely evaluated in various diseases^1,2,5^ since 2007, when the first report showed the cytoprotective function of H_2_ as an effective antioxidant that eliminated cytotoxic oxygen radicals^7,42^. Thousands of studies in different disease models ranging from acute to chronic diseases, including cancer, support the hypothesis that H_2_ has a wide spectrum of activity. Unlike conventional drugs, a specific primary target has not yet been identified for H_2_, which would explain its broad efficacy in different diseases. H_2_ showed great success in controlling various diseases^1,2,5^ and is being evaluated for clinical applications in healthy people to improve performance and body condition^13,20,23^. However, it remains unknown how H_2_ performs under healthy conditions. Therefore, this study aimed to fill this gap by providing molecular data to help further our understanding of the biological activities of H_2_.

As we previously reported, long-term H_2_ intervention significantly affects physiological and biochemical parameters of healthy rats, with a significant alteration noted for liver biomarkers^24^. Moreover, no significant changes were observed for indicators of oxidative stress such as superoxide dismutase (SOD), catalase (CAT), and malondialdehyde (MDA) (Supplementary Figure 1C-E). Given that liver is the central and metabolic hub that connects most organs^33^ and has the highest H_2_ accumulation rate^4,9^, we have focused our study for this time on the molecular alteration in liver that can result from H_2_ intervention. Furthermore, we have not observed any significant change for the global liver transcriptomes or metabolites between HRW and HI; therefore, the data in the present manuscripts are presented and discussed without any discrimination between the two H_2_ groups.

Analysis of liver gene expression of healthy rats collected at the end of 6 months of H_2_ intervention revealed a significant alteration of lipid metabolism pathways, confirming the observation of previous reports, which had shown the ability of H_2_ to modulate lipid profile and functions^43–45^. Like in short-term studies of H_2_ interventions, we have found long-term exposure to H_2_ to reduce the serum level of total cholesterol^24^. This effect appears to be likely extended to other sterol lipids, as we observed a decrease in the level of several other sterol lipids, including glycocholic acid, ursodeoxycholic acid, turochenodeoxycholic acid, and corticosterone from the metabolomic study. The level of low-density lipoprotein cholesterol (LDL-C) was reduced, and no significant changes were observed for high-density lipoprotein cholesterol (HDL-C) in HRW-treated rats, consistent with previous observations^24,43–45^. Meanwhile, we found that HDL-C is significantly reduced by inhalation of H_2_. The biological relevance of this alteration would require further investigation, as HDL-C can sometimes become bad cholesterol and increase the risk of atherogenesis^46–48^. Our data, like others, confirmed that H_2_ possesses lipid metabolic regulatory activities. Moreover, the absence of accumulation of lipids in the liver (Supplementary Fig. 3), as a consequence of the low serum cholesterol level, makes H_2_ a relevant therapeutic option for people with disorders of lipid metabolism.

It is still unclear how H_2_ fulfills the regulatory function of lipid metabolism. In the present study, we found that in addition to decreasing the level of serum and liver sterol lipids, long-term H_2_ treatment reduced the level of liver fatty acyls by probably inducing their transport to mitochondria for oxidation, as evidenced by the increase in the level of mitochondrial fatty acid transporters, acylcarnitines. These results, together with the increase in the level of acetoacetate, a marker of ketogenesis in the liver^30,31^, suggest that H_2_ induces oxidation of fatty acids and ketogenesis. Circulating epinephrine regulates the activity of hormone-sensitive lipase that controls the rate of ketogenesis^31,34,35^. Hormone-sensitive lipase is responsible for mobilizing free fatty acids from adipose tissues to serve as substrate for ketogenesis^31,49,50^. Considering the substantial loss of adipose tissue and the increased level of hormone-sensitive lipase and epinephrine in H_2_-treated rats, H_2_ appears to promote lipolysis in adipose tissue, resulting in the release of free fatty acids which are transported and converted to ketone bodies in the hepatic mitochondria.

The transcriptional profile of rats treated with H_2_ showed a significant up-regulation of genes enriched in the amino acid catabolic process, consistent with the reduced level of amino acids involved in the glycine, serine, and threonine metabolic pathways and the arginine and proline metabolic pathways. We also found that H_2_ treated rats have purine nucleotides and carbohydrates biosynthesize pathways activated. Knowing that the main use of amino acid breakdown is to provide building blocks for the synthesis of nitrogen-based compounds, protein synthesis, and as metabolic fuels^51^, it is possible that H_2_ triggered the catabolism of these amino acids to support ketogenesis, nucleotide, and carbohydrates biosynthesis reactions.

Although a specific target has not yet been identified to support the therapeutic effects of H_2_, there is a consensus on its antioxidant properties. Cells respond to oxidative stress by modulating the redox system, which depends on the availability of reducing agents, including NADPH. NADP is essential for generating NADPH to provide the reduction power that maintains redox homeostasis and regulates cell metabolism^36,37^. In this study, we found that H_2_ significantly decreases the level of NADP, which was accompanied by the activation of NADPH production pathways such as PPP. The level of NAD, which can also be generated by NADP dephosphorylation^52,53^, remained unchanged (Supplementary Figure 2), suggesting that H_2_ induces NADP reduction into NADPH. Type 2 cytochrome P450s located in the endoplasmic reticulum use cytochrome P450 oxidoreductase (POR) as their redox partner. POR transfers electrons from NADPH to CYP450s in reactions that result in the generation of NADP^41^. In the present study, NADP levels decreased after H_2_ treatment. Furthermore, the genes that encode differentially expressed POR enzymes and enzymes such as Hmgcs1, Hmgcr, Dhcr24 upstream of the Cyp51-dependent cholesterol synthesis pathway were negatively regulated, except for Cyp8b1, Cyp7b1, which are involved in cholesterol metabolism into bile acids^54^. The enzymatic activity of Hmgcr and CYP7A1 measured in liver homogenates from experimental rats, showed no significant differences (Supplementary Figure 4). Furthermore, analysis of the network of interaction of genes and metabolites connects NADP to metabolic pathways significantly affected by the long-term H_2_ intervention. These findings indicate that H_2_ must modulate biological pathways that involve the reduction of NADP to NADPH, and suggested that investigating biological pathways that require NADP as electron acceptors, such as PPP, could help identify the mechanisms to support the metabolic alteration observed in the liver during prolonged use of H_2_.

In conclusion, long-term use of H_2_ appears to trigger lipid and amino acid catabolism in the liver to provide energy and building blocks for purine nucleotides and carbohydrates biosynthesize reactions by modulating pathways involving the redox couple NADP / NADPH. This study is the first to provide molecular data to help better understand the biological effect of H_2_ on liver metabolism under healthy conditions. Furthermore, the significant impact of H_2_ on lipid metabolism observed in this study provides a context to recommend its use in lipid metabolism disorders.

## Supplementary Information

Supplementary Information 1.

Supplementary Information 2.

Supplementary Information 3.

Supplementary Figure 1.

Supplementary Figure 2.

Supplementary Figure 3.

Supplementary Figure 4.

## Abbreviations

ALT: Alanine aminotransferase
TBA: Total bile acid
AST: Aspartate aminotransferase
HRW: Hydrogen-rich water
HI: Hydrogen inhalation
BAT: Brown adipose tissue
WAT: White adipose tissue mass
CTRL: Control
