# Supplementary material for: Long-term and daily use of molecular hydrogen induces reprogramming of liver metabolism in rats by modulating NADP/NADPH redox pathways
Source: Sci Rep. 2022 Mar 10;12:3904. doi: 10.1038/s41598-022-07710-6 (PMC8913832; doi:10.1038/s41598-022-07710-6)
Supplement: Supplementary file 4 — Supplementary Figure 1. [file 41598_2022_7710_MOESM4_ESM.pdf]

Long-Term and Daily Use of Molecular Hydrogen Induces Reprogramming of Liver Metabolism in Rats by Modulating NADP/NADPH Redox Pathways

Yao Mawulikplimi Adzavon[1,2#], Fei Xie[1,2#], Yang Yi[1,2], Xue Jiang[1,2], Xiaokang Zhang[1,2], Jin He[1,2], Pengxiang Zhao[1,2], Mengyu Liu[1,2], Shiwen Ma[1,2], Xuemei MA[1,2*]

Supplementary figure 1

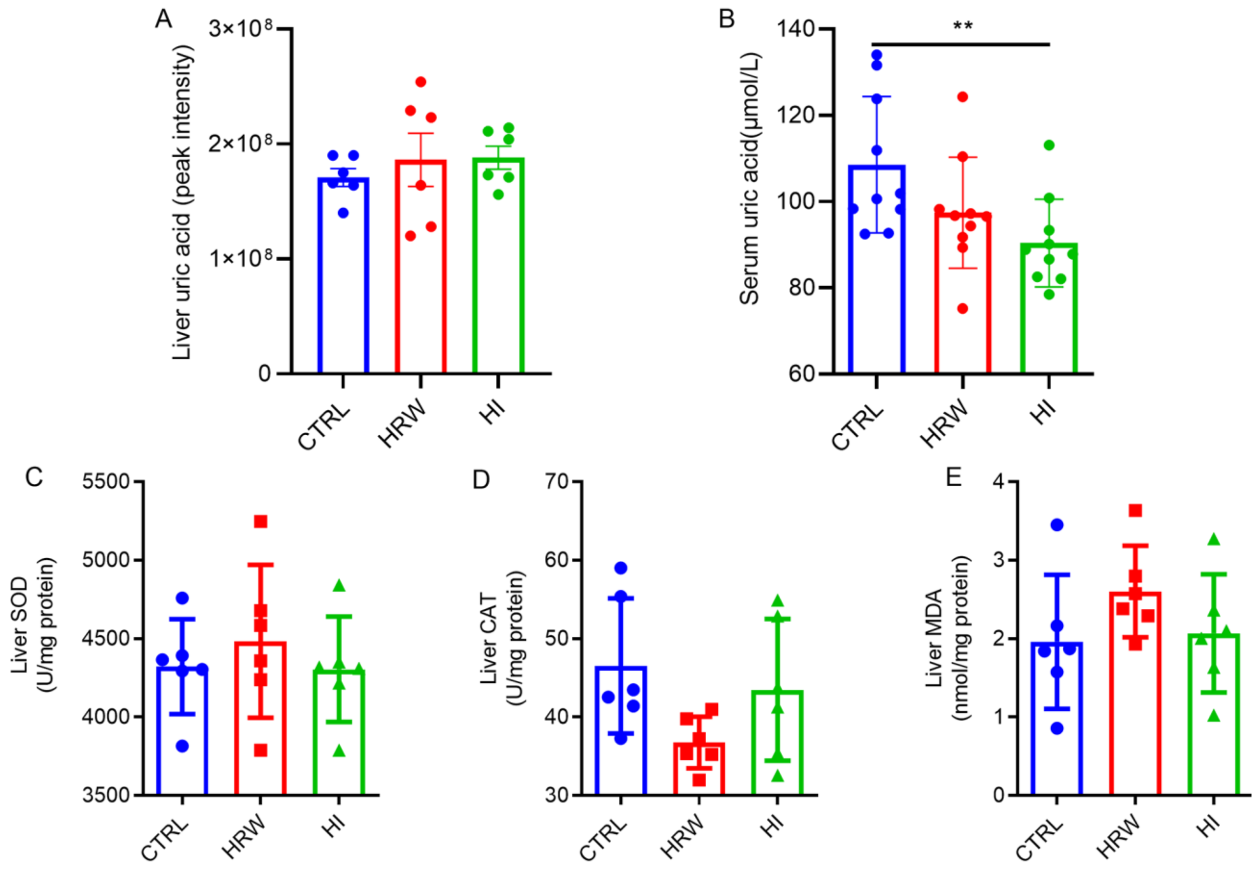

Effect of long-term H_2 intervention on the level of Uric acid analyzed by metabolomics in the liver (A) and by ELISA in serum (B) of healthy rats. (C-E) Indicators of oxidative stress in liver of H_2-treated rats compared to untreated rats: enzymatic activity of SOD (superoxide dismutase), CAT (catalase), and the concentration of MDA (Malondialdehyde) was measured by ELISA in liver lysates. Data are presented as Mean ± SEM. * p-value < 0.05; ** p-value <0.01; *** p-value < 0.001
