# Supplementary material for: Long-term and daily use of molecular hydrogen induces reprogramming of liver metabolism in rats by modulating NADP/NADPH redox pathways
Source: Sci Rep. 2022 Mar 10;12:3904. doi: 10.1038/s41598-022-07710-6 (PMC8913832; doi:10.1038/s41598-022-07710-6)
Supplement: Supplementary file 5 — Supplementary Figure 2. [file 41598_2022_7710_MOESM5_ESM.pdf]

Long-Term and Daily Use of Molecular Hydrogen Induces Reprogramming of Liver Metabolism in Rats by Modulating NADP/NADPH Redox Pathways

Yao Mawulikplimi Adzavon[1,2#], Fei Xie[1,2#], Yang Yi[1,2], Xue Jiang[1,2], Xiaokang Zhang[1,2], Jin He[1,2], Pengxiang Zhao[1,2], Mengyu Liu[1,2], Shiwen Ma[1,2], Xuemei MA[1,2*]

Supplementary figure 2

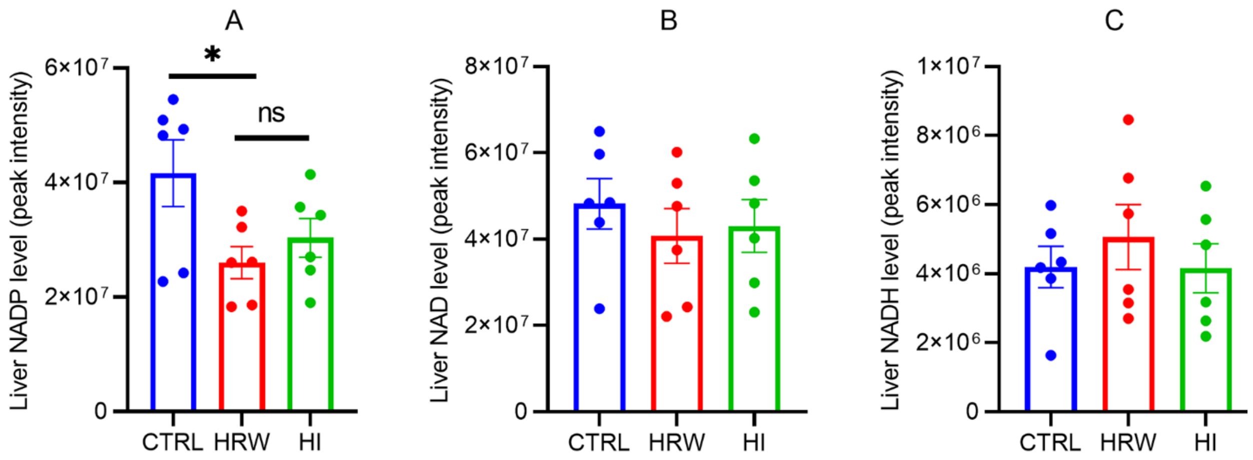

(A) Peak intensity of NADP data from the metabolomics analysis related to Fig 3B. Change in the level of NAD (B) and NADH (C) analyzed by metabolomics in the liver of rats exposed daily to H_2 for 6 months. Data are presented as Mean ± SEM. * p-value < 0.05; ** p-value <0.01; *** p-value < 0.001
