# Supplementary material for: Long-term and daily use of molecular hydrogen induces reprogramming of liver metabolism in rats by modulating NADP/NADPH redox pathways
Source: Sci Rep. 2022 Mar 10;12:3904. doi: 10.1038/s41598-022-07710-6 (PMC8913832; doi:10.1038/s41598-022-07710-6)
Supplement: Supplementary file 6 — Supplementary Figure 3. [file 41598_2022_7710_MOESM6_ESM.pdf]

Long-Term and Daily Use of Molecular Hydrogen Induces Reprogramming of Liver Metabolism in Rats by Modulating NADP/NADPH Redox Pathways

Yao Mawulikplimi Adzavon[1,2#], Fei Xie[1,2#], Yang Yi[1,2], Xue Jiang[1,2], Xiaokang Zhang[1,2], Jin He[1,2], Pengxiang Zhao[1,2], Mengyu Liu[1,2], Shiwen Ma[1,2], Xuemei MA[1,2*]

Supplementary figure 3

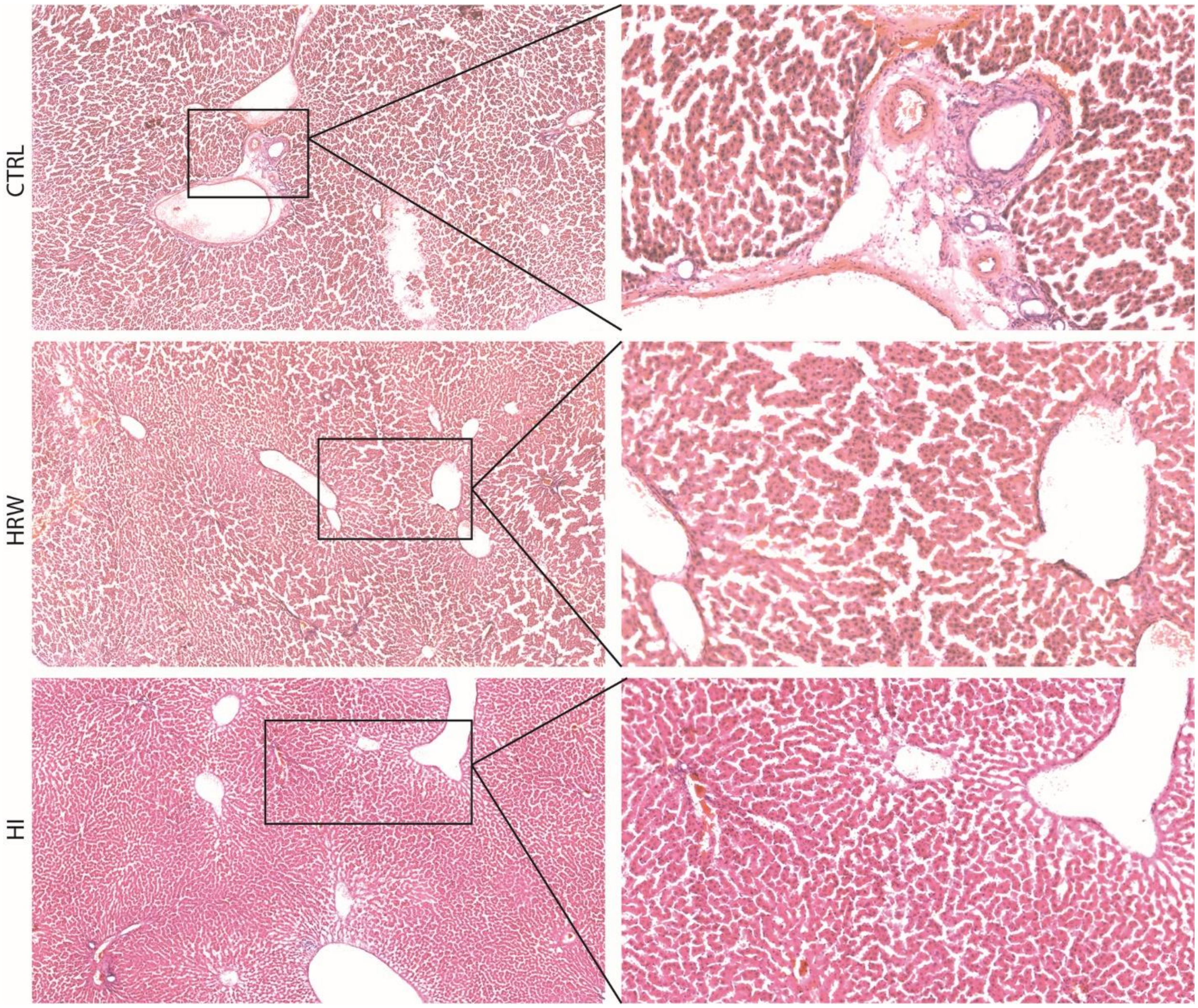

H&E staining of liver biopsies of rats untreated and H_2-treated for 6 months, showing no signs of liver steatosis.
